# Chemical Synthesis of Sulfated Yeast (*Saccharomyces cerevisiae*) Glucans and Their In Vivo Antioxidant Activity

**DOI:** 10.3390/molecules22081266

**Published:** 2017-07-28

**Authors:** Hua Zhang, Jing Zhang, Ziluan Fan, Xintao Zhou, Lin Geng, Zhenyu Wang, Joe M. Regenstein, Zhiqiang Xia

**Affiliations:** 1 Department of Food Science and Engineering, School of Chemistry and Chemical Engineering, Harbin Institute of Technology, Nangang District, Harbin 150090, China; yyzanlzg@163.com (J.Z.); ZhouXT2016@163.com (X.Z.); neauxiaoxue@163.com (Z.X.); 2 MIIT Key Laboratory of Critical Materials Technology for New Energy Conversion and Storage, School of Chemistry and Chemical Engineering, Harbin Institute of Technology, Nangang District, Harbin 150001, China; 3 School of Materials Science and Engineering, Harbin Institute of Technology, Nangang District, Harbin 150001, China; genglin@hit.edu.cn; 4 School of Forestry, Northeast Forestry University, Xiangfang District, Harbin 150040, China; fzl_1122@163.com; 5 Department of Food Science, Cornell University, Ithaca, NY 14853-7201, USA; jmr9@cornell.edu

**Keywords:** yeast, yeast glucans (YG), sulfated yeast glucans (SYG), *Saccharomyces cerevisiae*

## Abstract

The effects of sulfation of yeast glucans was optimized using response surface methodology. The degree of sulfation was evaluated from 0.11 to 0.75 using ion-chromatography. The structural characteristics of SYG (sulfation of yeast glucans) with a DS = 0.75 were determined using high-performance liquid chromatography/gel-permeation chromatography and finally by Fourier transform infrared spectrometry. The SYG had lower viscosity and greater solubility than the native yeast glucans, suggesting that the conformation of the SYG had significantly changed. The results also showed that SYG had a significantly greater antioxidant activity in vivo compared to native yeast glucans.

## 1. Introduction

Glucans from different sources have different types of glycosidic bond. Yeast glucans are polysaccharides that are mainly linked by β-glycosidic bonds (1,3) and (1,6). Vetvicka et al. [1] found that oral administration of β-glucans stimulated hematopoiesis in radiation-treated mice. In addition, β-glucan has been postulated to modulate antioxidant enzyme activity (SOD) as well as to inhibit lipid peroxidation in studies concerning rat or rabbits [2]. The grass carp infected with the bleeding virus (GCHV) was treated with β-glucan, superoxide dismutase (T-SOD) and catalase (CAT) activity increased to a high level in serum [3]. β-Glucan (from fungi *Pleurotus ostreatus*) increased SOD and CAT activity in rat erythrocytes [4,5]. Other studies have shown that biological activities of polysaccharides are increased by chemical modification such as sulfation [6]. The sulfated polysaccharides have been shown to have biological activities, such as anti-coagulant [7,8], anti-virus [9], anti-oxidant [10,11] and antitumor activity [12,13]. Therefore, sulfation can be used to improve the biological activity of some polysaccharides [14].

Many reagents have been used to sulfate polysaccharides such as sulfuric acid, chlorosulfonic acid (CSA)–pyridine, sulfur trioxide–pyridine (SO_3_·Py), and sulfur trioxide–dimethylacetamide (DMF) [15]. CSA-DMF was used to synthetize sulfate polysaccharides which have high yields and high sulfation degrees (DS) compared with other sulfation reagents. The ratio of the DMF to CSA, reaction temperature and reaction time are important factors when using this method. 

Many other factors may affect the desired result. A response surface method (RSM) can therefore be an effective tool to obtain the desired outcome with a minimum number of experiments. RSM has been used widely in scientific research, such as on cellulose nanocrystals [16]. Therefore, this method will be used to optimize the preparation of sulfated yeast glucans (SYG). In this study, SYG with various DS was prepared using the CSA–DMF method. In the present investigation on structural characterization, antioxidant properties of the SYG have been carried out and reported here.

## 2. Materials and Methods

### 2.1. Materials and Reagents

Angel Yeast Co., Ltd. (Yi Chang City, Hubei Province, China) supplied the yeast glucans from *Saccharomyces cerevisiae*. The T-series Dextran’s were obtained from Agilent (Beijing, China). The chlorosulphonic acid (HClSO_3_), *N*,*N*-dimethyl formamide (DMF), formamide (FA), and HCl were of analytic reagent grade and obtained from Sigma Chemical Co. (St. Louis, MO, USA). All solutions were prepared in double-distilled water (DDW).

### 2.2. Experimental Design 

Yeast β-glucan was produced from *Saccharomyces cerevisiae*. *Saccharomyces cerevisiae* were used as the raw material for the extraction followed by treatment with acid and alkali and spray driedto produce the yeast β-glucans (Angel Yeast Co. Ltd., Yi Chang City, Hubei Province, China). The sulfating of YG was done using the method of Wang et al. [17] with modifications. The sulfating reagent, DMF-SO_3_, was obtained by dropping 50 mL HClSO_3_ into 300 mL of DMF while being cooled in an ice-water bath. Dry YG (1.0 g) was added to 40 mL FA, and the mixture was stirred using a magnetic stirrer at 50 °C for three h to disperse into the solvent. Then 10 mL of DMF-SO_3_ reagent was added. After three h, the mixture was cooled to 25 °C and precipitated with 75% ethanol for 24 h. Following this procedure, the precipitate was washed three times with 60% ethanol and then dissolved in 100 mL of DDW. The solution was neutralized with 1.0 mol/L NaOH solution and dialyzed against tap water for 48 h and DDW for 24 h using 3500 Da Mw cutoff dialysis membranes according to the manufacturer (Spectrum Medical Industries, Inc., Los Angeles, CA, USA). The SYG obtained was quickly frozen at −40 °C and dried for 48 h using a freeze dryer (LGJ-25C, Four-Ring Science Instrument Plant Beijing Co., Ltd., Beijing, China).

The response surface method (RSM) is an efficient way to determine the best conditions with a minimum number of experiments. The ratio of the DMF-SO_3_ (X_1_), the temperature (X_2_), and the reaction time (X_3_) were chosen as independent variables. The three variables were assessed using a Box-Behnken design with three factors, three levels, and three replicates at the central point. In this way, the total number of experiments with the three variables was 17. Based on a set of single factor experiments (data not shown), X_1_ (1:4, 1:6 and 1:8), X_2_ (40, 50 and 60 °C) and X_3_ (2, 2.5 and 3 h) were determined as the critical levels that had significant effects on the DS of SYG. The model proposed for the response Y (DS) is given below.

*Y* = b_0_ + b_1_X_1_ + b_2_X_2_ + b_3_X_3_ + b_4_X_1_ X_2_ + b_5_ X_1_X_3_ + b_6_ X_2_X_3_ + b_7_X_1_^2^ + b_8_X_2_^2^ + b_9_X_3_^2^

where X_1_: Ratio of DMF: HClSO_3_; X_2_: Temperature (°C); and X_3_: Time (h). *Y* was the predicted response (the DS), b_0_ was the constant and terms, b_1_, b_2_ and b_3_ were the coefficients of the linear terms, b_4_, b_5_ and b_6_, were the coefficients of the interaction effects; and b_7_, b_8_ and b_9_ were the coefficients of the quadratic effects. 

The adequacy of the polynomial model was evaluated using multiple coefficient of determination: R^2^. The significance of each coefficient was determined using its *F* and *p* values. The software Design-Expert^®^ 7.0.0 trial version (State-Ease Inc., Minneapolis, MN, USA) was used to obtain the coefficients of the quadratic polynomial model and evaluate the results.

### 2.3. Degree of Sulfation (DS)

To determine DS, the method of Zhang et al. [10] was used. SYG in a screw-cap tube was hydrolyzed by adding 4 mL 1.0 mol/L HCl at 100 °C for 6 h. The hydrolysis liquid was volatilized with a nitrogen stream at 45 °C. All standards and samples were analyzed using an ion-chromatography (IC) system which consisted of a Dionex ICS-3000 (Bannockburn, IL, USA) equipped with a dual piston pump, degasser, suppressor, column heater, autosampler, and conductivity detector. Data collection and analysis were done using Chromeleon software, version 6.8 (Thermo Fisher Scientific Inc., Waltham, MA, USA) supplied with the system. Separation was done on a Dionex IonPac AS11 analytical column (4 × 250 mm) with an IonPac AG11 guard column (4 × 50 mm). The mobile phase was 30 mmol/L KOH delivered isocratically at a flow rate of 1.0 mL/min. The column temperature was 30 °C. The injection volume was 20 μL and detection was done using suppressed conductivity to eliminate interference from the mobile phase ions. Suppression was achieved with an anion suppressor (ASRS 300 4 mm) from Dionex. Suppression current was set at 124 mA. The pool temperature was 35 °C.

A linear regression line was obtained: *y* = 4.15*x* − 0.0518 (R^2^ = 0.9998) using sodium sulfate powder as the standard. Calibration ranges of SO_4_^2−^ Ion are from 1 to 100 (mg/L). The DS was calculated using a previously reported method that incorporated the following equation [10].

*DS* = (1.62 × %*S*)/(32 − 1.02%*S*) (1)

where %*S* is the mass fraction of sulfur in the sample.

### 2.4. Molecular Mass Distribution

The molecular weight (MW) of SYG was measured using high performance gel-permeation chromatography (HPGPC) on an Agilent 1100 HPLC system (Agilent, Santa Clara, CA, USA) equipped with a differential refractive index detector (RID) and automatic sample injector using the method of Zhang et al. [10]. All separations were done using a PL Aquagel-OH Mixed column (300 mm × 7.5 mm i.d. × 8 μm, Agilent). The retention times of the Dextran T series standards (106, 194, 620, 1470, 4120, 11,800, 25,800, 58,400, 125,000, 465,000, 965,000, and 1,250,000 Da according to the manufacturer) were plotted against the logarithms of their corresponding average MW. All spectra were acquired by the ChemStation software system (A.09.01, G2170AA, A.02.01, G2182AA, Agilent Technologies, Palo Alto, CA, USA) that came with the system. The mobile phase was 0.02% NaN_3_ with a flow-rate of 1.0 mL/min. A 50 μL sample was passed through a 0.45 μm cellulose acetate filter (Dong Kang Co. Tianjin, China) prior to injection.

### 2.5. Solubility

The solubility of SYG was measured using Xie et al.’s method [18]. 2 g SYG (denoted as *M*) was weighed and added to 10 mL of deionized water and shaken for 20 min. After the completion of the oscillation, the mixture was centrifuged at 3500× *g* gravity for 20 min, and the supernatant was dried at 105 °C. The weight drying powder was written down *M*_1_.

The solubility is calculated as follows:

*The solubility%* = *M*_1_*/M ×* 100 (2)

### 2.6. Viscosity

Capillary viscometry was performed using an Ubbelohde vis-cometer in accordance with the method of Zhao et al. with minor modifications [19]. The elution time of different concentrations of SYG (*t*_1_) and the elution time of the solvent (*t*_0_) were determined by a capillary viscometer. The intrinsic viscosity of polysaccharide was calculated according to the following formula:

*η_sp_= t*_1_*/t*_0_ − 1 (3)

### 2.7. Infrared Spectroscopy

A SYG sample for FT-IR was obtained by grinding a mixture of polysaccharide with dry KBr and then pressing it into a mold The IR spectra were recorded on a Spectrum one FT-IR spectrometer (PerkinElmer, Norwalk, CT, USA) and run in the 4000–400 cm^−1^ region at a resolution of 4 cm^−1^ [20]. The total number of scans recorded was 16. The FT-IR spectrum was used to analysis the possible functional groups and bond types and their changes.

### 2.8. SYG’s Effect on Radiation-Induced Injury of Mice

Forty Kunming laboratory mice (half male and half female) of the most commonly used out-crossed mouse in China derived from Swiss mice were used. They weighed 18–22 g and were approximately 28 days old. They were purchased from the Laboratory Animal Center of the Second Hospital of Harbin University (Harbin, Heilongjiang Province, China). Before the start of the research, groups of 10 sex-separated mice were kept in stainless steel cages under a constant 12 h light/dark cycle at 25 °C. They were fed normal commercial diets and water *ad libidum*. All mice were allowed to acclimatize to the laboratory environment for a week before the study.

#### 2.8.1. Experimental Design and Radiation Exposure

The 40 mice were randomly assigned at the end of the week of acclimatization to one of the four following treatment groups: untreated control, treated control (positive control), YG and SYG. All mice, except the untreated controls, were exposed to whole-body radiation on day 15 after administration intra-gastrically of YG or SYG (50 g/kg body weight per day every day for both treatments). Both controls were administered intra-gastrically with the same volume of DDW every day. Radiation was done at the Maize Research Institute of Heilongjiang Academy of Agricultural Sciences (Harbin, China) using their equipment. All mice, except those from the normal control group, were irradiated for 6¼ min with a single 6.0 Gy dose delivered using a ^60^Co tube operated with an output of 1.0 Gy/min at 160 cm. Each mouse was placed in a separate plastic container (20 × 20 × 100) immediately after irradiation.

#### 2.8.2. Body Weight and Immune Organ Indexes

The mice were monitored daily for growth and mortality. The body weight of each mouse was measured daily. One day after receiving radiation, all mice were slaughtered. To analyze the spleen and thymus indexes, these organs were immediately removed surgically and weighed. These procedures were all done following the ethical standards of the Animal Welfare Committee of Heilongjiang Province, China. The thymus and spleen indexes were calculated using the following previously described formula [21,22].

*Thymus or spleen index* = (*weight of thymus or spleen/body weight*) × 100% (4)

#### 2.8.3. Transmission Electron Microscope (TEM) 

To observe apoptosis and the cell morphology of the splenic lymphocytes in the mice, the method of Dong et al. was used [23]. After flushing with normal saline, the spleen was cut into small blocks (8–10) using sharp scissors; each <1 mm^3^; then 5% glutaraldehyde solution was added and the treated spleen was then refrigerated at 4 °C for 2 h. The samples were washed in pH 7.2 PBS for 1 h, and post-fixed in 2% osmic acid at 4°C for 1 h at 4 °C. After post-fixing for 1.5 h, the samples were washed three times with cold PBS for 15 min each time. The samples were stained with 2% aqueous uranyl acetate solution for 2 h. Subsequently, the samples were dehydrated using an ethanol gradient of 50, 70, 80, 90 and 95%, each time of dehydration was 15 min, and then replaced with acetone, embedded in epoxide resin (Blue Star, Wuxi, Jiangsu, China) overnight. The dehydrated samples were dried at 60 °C for 72 h and prepared as semi-thin sections. Ultra-thin sections, 60–100 nm thick, were collected using a microtome (PerkinElmer, Norwalk, CT, USA). The sections were then placed on copper grids, stained with uranyl and lead citrate and viewed in a TEM (H-500, Hitachi, Tokyo, Japan) under high vacuum at an acceleration voltage of 100 kV and image magnifications of 8000~20,000×.

#### 2.8.4. Splenocyte Proliferation Index

On the 1st day after irradiation, the spleens were ground with a mortar and pestle and then the pestle was used to push the spleens through a 200-mesh filter (Xin Xing Co., Harbin, Heilongjiang, China) and washed with 5 mL pH 7.4 PBS. The number of viable cells was determined using the trypan blue (Sigma Aldrich, St. Louis, MO, USA) exclusion test. The spleen cell suspension was washed three times in PBS and the cell concentration was adjusted to 5 × 10^6^/mL in 96-well plates (100 μL/well) in RPMI-1640 culture medium with 10% fetal calf serum (Gibco, Paisley, UK). Data were averaged for three independent experiments run in duplicate. Concanavalin A (Sigma Aldrich, St. Louis, MO, USA) (CoA, 5 μg/well) was added to the wells. Culture medium was added to negative control wells in place of Con A. The cells were cultivated in 5% CO_2_ (purity of 99.9%, Harbin LiMing Gas Co. Harbin, Heilongjiang, China) at 37 °C for 44 h using an incubator (Class 100, Thermo heap, Thermo Fisher Scientific Inc., Rochester, NY, USA), and then 5 mg/mL methylthiazolyl tetrazolium salt (MTT (Sigma Aldrich), 10 μL/well) was added for 4 h. To enable the purple crystals to dissolve completely, the cell suspension was removed and re-dissolved with DMSO (100 μL/well). The optical density (*OD*) was determined at 570 nm (T6 New Century UV/Vis spectrophotometer, Puxi Instrument Co., Ltd., Beijing, China). The *OD* value can indirectly reflect the number of living cells [24]. In a certain cell number range, the amount of MTT crystal formation is proportional to cell number. The proliferation index was calculated using the following described formula:

*The proliferation index* = *(OD_experimental well_* − *OD_control well_/ OD_control well_) ×* 100% (5)

#### 2.8.5. Radiation-Induced Mice Biochemical Changes

Malondialdehyde (MDA) level, catalase (CAT) activity, and superoxide dismutase (SOD) activity in the spleen of each of the irradiated mice was then measured using commercial kits following the manufacturer’s instructions (Nanjing Jiancheng Bioengineering Institute, Nanjing, Jiangsu, China).

### 2.9. Statistical Analysis

The results were analyzed using STATISTICA for Windows Version 8.0 (StatSoft Inc. 2007, Tulsa, OK, USA). Significant differences between two means were determined by Tukey’s test. A *p*-value < 0.05 was regarded as significant.

## 3. Results and Discussion

### 3.1. Preparation of SYG 

Due to the strong dehydration ability of chlorosulfonic acid with the reaction conditions used, it was possible that the polysaccharides with hydroxyl groups were dehydrated. Therefore, the degradation of polysaccharide with sulfation probably involved both de-hydrolysis and hydrolysis.

**Figure 1:**
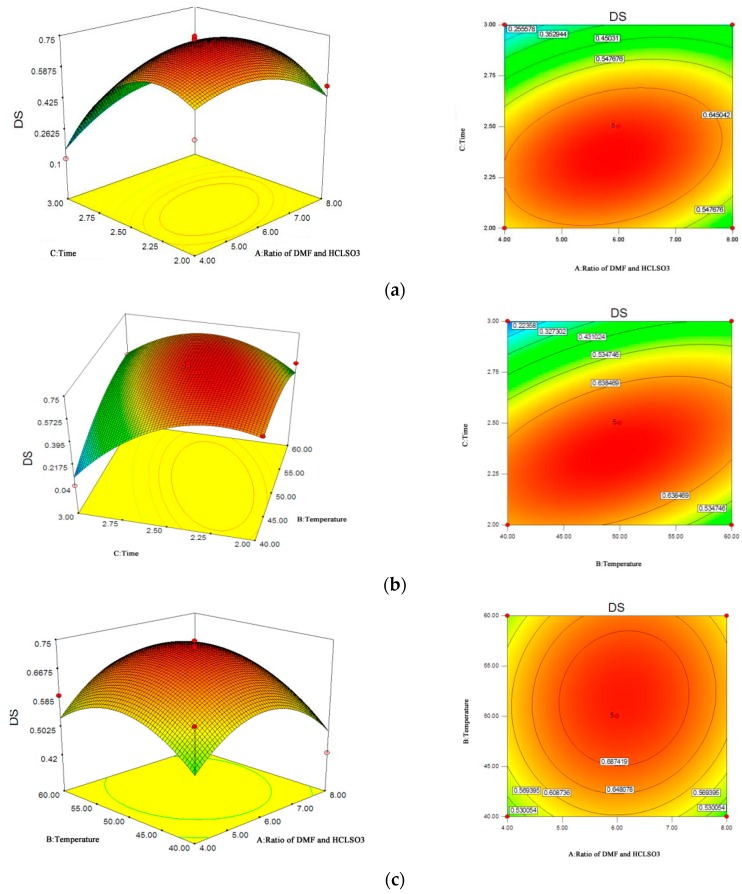
Response surface plots. (**a**) Effect of the ratio of DMF (*N*,*N*-dimethyl formamide) and HClSO_3_ and reaction time on DS of SYG; (**b**) Effect of reaction temperature and reaction time on content of DS of SYG; (**c**) Effect of reaction temperature and ratio of DMF and HClSO_3_ on DS of SYG.

Figure 1a shows the effect of time and the ration of DMF–HClSO_3_ on the DS of the SYG. The curved surface in this illustration shows a steep slope that gradually and slightly decreases. This outcome established that in the experimental range, the DS increased with the increase of time. However, the amplitude of the increase slowed. Nevertheless, as the ratio of DMF–HClSO_3_ increased, the DS first showed an increase and then a decrease. The interaction between time and DMF-HClSO_3_ ratio was not significant. 

Figure 1b shows the effect of temperature and time on the DS of the SYG. When the time was set, and the temperature increased, the contour line of the interaction contours of time and reaction temperature indicated that the DS of the SYG rapidly increased. This suggested that the temperature significantly (*p* < 0.05) influenced the DS. At the same temperature, the DS of the product increased with time, and the impact of time was greater than that of the temperature. That is, the increase of both temperature and time increased DS significantly (*p* < 0.05).

Figure 1c shows the effects of temperature and the DMF-HClSO_3_ ratio. At a fixed DMF–HClSO_3_ ratio, increased temperature increased DS rapidly. The interaction between the ratio of DMF–HClSO_3_ and the temperature did not impact the DS significantly, indicating a reduced effect on the DS when the ration of DMF–HClSO_3_ was changed from 4:1 to 8:1. The DS was enhanced with increasing temperature and reached a peak value at 50 °C.

### 3.2. Optimization of Synthesis Parameters with SYG

#### 3.2.1. Model Fitting

Table 1 shows the ‘value of the response’ (DS) for different experimental combinations for the three variables including their interactions. The stability and inherent variability of the synthesis system were examined by completing three center-point runs. 

#### 3.2.2. Model Fitting

The regression equation obtained was:

Y = −3.232 + 0.1087X_1_ + 0.02566X_2_ + 2.580X_3_ + 6.930 × 10^−^^4^X_1_X_2_ + 0.08368X_1_X_3_ + 0.02559X_2_X_3_ − 0.02888X_1_^2^ − 9.074×10^−4^X_2_^2^ − 0.9241X_3_^2^

A positive or negative coefficient indicates a synergistic or antagonistic effect, respectively. The results of the ANOVA testing of the model are shown in Table 2. A model *F*-value of 4.75 suggests that the model is significant while a *p*-value less than 0.0001 indicates that the model terms are significant, while the lack of fit was not significant (*p ≥* 0.05). The multiple coefficient of correlation (R = 0.9930) showed a high agreement between experimental and predicted values of the DS. As shown in Table 2, the cross-product coefficients (X_2_X_3_) were significant as was X_3_; whereas X_1_, X_1_X_2_, X_1_X_3_ and X_2_ were all insignificant (*p ≥* 0.05). The *F* values indicated that the order of factors influencing DS were time > temperature > the ration of DMF-HClSO_3_, and the order of the interaction effects was X_2_X_3_ > X_1_X_2_ > X_1_X_3_.

#### 3.2.3. Verification of Results

The optimal conditions for maximizing the DS of SYG was predicted from the model as: the ratio of the DMF and the HClSO_3_: 5.87:1, time: 2.35 h, and temperature: 49.5 °C to give a predicted DS of 0.72 ± 0.02. The predicted point was experimentally verified using the optimum conditions: the ratio of the DMF and the HClSO_3_ was 5.9:1, time: 2.4 h, temperature: 49.5 °C showing the model was acceptable.

### 3.3. Determination of the DS

The calculations showed that the DS of the SYG was 0.75, which was in accord with the sulfate group content obtained with IC.

### 3.4. Molecular Mass Distribution 

The HPGPC chromatogram of YG and SYG had peaks in the 4–10 min region. The retention time of the YG and SYG were plotted on the same graph. A linear regression standard curve of log MW versus GPC retention time was calculated. The linear regression equation was obtained: Log MW = 0.668 Tr + 8.84 (R^2^ = 0.9713) where Tr equals the retention time. When referenced to the standard dextrans, the MW of the YG and the SYG were 8.51 × 10^6^ Da and 1.29 × 10^6^ Da, respectively. Furthermore, the molecular mass distribution of the YG was narrower than that of the SYG. These experimental results confirmed that the average MW of the SYG was less than that of the YG, suggesting that some hydrolysis was also taking place. The SYG’s viscosity and solubility were 0.947 ± 0.001 × 10^−4^ cSt/s and 2.15 g/100 g at 25 °C, respectively. The solubility of the SYG significantly increased more than 28 times compared with YG.

### 3.5. Fourier Transform Infrared (FT-IR)

**Figure 2:**
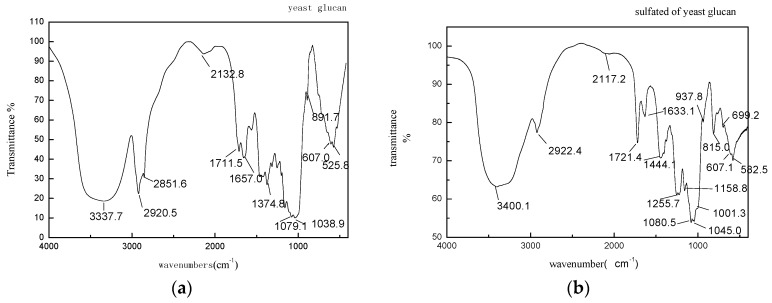
FTIR spectra. (**a**) YG; (**b**) SYG.

FT-IR made it possible to analyze polysaccharide structures such as monosaccharide types, glucosidic bonds, and functional groups [25]. In all spectra (Figure 2), the band in the region of 3338 and 3400 cm^−1^ of the YG and the SYG corresponded to the hydroxyl stretching vibration of the polysaccharide and at 2921 and 2922 cm^−1^ corresponded to a weak C-H stretching vibration. Both indicated that the YG and the SYG were polysaccharides. By comparison with the YG, two characteristic absorption bands appeared in the FT-IR spectra of the sulfated derivatives (Figure 2b). One band in the region of 1256 cm^−1^ is believed to represent an asymmetrical S=O stretching vibration and the other at 815 cm^−1^ represents a symmetrical C–O–S vibration associated with the C–O–SO_3_ group. The data suggested that the SYG was successfully sulfated [25]. 

### 3.6. Effect on Radiation-Induced Change of Mice’s Body Weight and Immune Organ Indexes

When compared with the weight of the mice before radiation treatment on the 14th day, the average weight of the radiated mice decreased for the one day after radiation. However, the rate of decreased body weight between the groups showed no significant differences (Table 3). This outcome verified that the selected radiation dose for the radiated mice showed no acute toxicity.

The thymus and spleen are the most important immune organs in animals and as such are highly sensitive to radiation damage. Mice, when exposed to 6.0 Gy γ-ray, are prone to oxidative damage; this damage results in organ atrophy and organ-index decline.

As shown in Table 3, it was found that the spleen and thymus indexes in the irradiated group were lower than the untreated group (*p* < 0.05), suggesting a γ-ray induced decline in their immune function. Although only the SYG treatment significantly increased the thymus index compared to the irradiated group (*p* < 0.05), treatments with either the YG or the SYG increased the spleen and thymus indexes compared to the irradiated group. The YG and the SYG treatment showed that both effectively stimulated the immune system of the mice so that the radiation damage was less. Although the exact mechanism for the immune-stimulating activity was not studied directly, it is proposed that both YG and SYG may act by inducing a number of antioxidant enzymes (SOD, CAT).

### 3.7. Micro-Morphology of Splenic Lymphocytes

An animal’s body when exposed to radiation that exceeds the recommended safety limit will be subjected to oxidative stress injury, hematopoietic system dysfunction, immune dysfunction, cell DNA strand breaks, gene mutations, chromosomal recombination, cell transformation, and cell demise [26]. Both splenic hematopoiesis and immunity are highly sensitive to radiation. Exogenous γ irradiation can induce apoptosis, inhibit immune function and induce degenerative changes in cells and tissues, including necrosis from the direct effect of radiation. Alternatively, it can induce neurohumoral dysregulation leading to karyopyknosis, necrosis, nuclear fragmentation, nuclear fusion, nuclear and cytoplasmic vacuolar degeneration, and changes in organizational structure [27].

Transmission electron microscopy (TEM) can be used to look for subtle changes in tissues and cells, or at the macromolecular levels of sub-structures, such as nuclear morphology, and nuclear membrane and chromatin changes. During apoptosis, and when linked to changes in function and metabolism, TEM helps to see tissue radiation injury and protection mechanisms. The apoptosis and the cell morphology of the splenic lymphocytes observed in the mice were the same as described previously by Bodnarchuk et al. [26].

**Figure 3:**
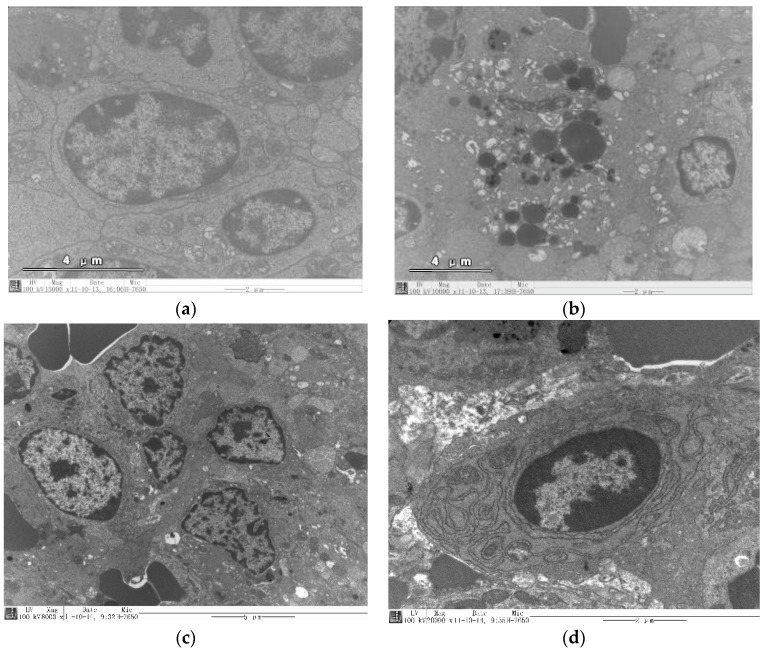
Microstructure analysis of mice spleen treated with YG (Yeast glucans) and SYG using Transmission Electron Microscopy. (**a**) Control; (**b**) Irradiated mice (**c**) Irradiated mice treated with YG; (**d**) Irradiated mice treated with SYG.

The large and small lymphocytes of the control group are shown in Figure 3. The shapes of splenic lymphocytes are mostly round or oval; some are shallow/concave shaped and have abundant organelles, which is consistent with Gao et al.’s findings [28]. Some show the nucleolus, complete nuclear membrane, cell membrane, and a small amount of cytoplasm. The cytoplasm is rich in ribosomes with some mitochondria, mitochondrial membranes with a continuous ridge, and with a Golgi complex (Figure 3a).

When assessed, the irradiated group’s structure and the spleen were still intact. There were more apoptotic cells in the white pulp and marginal regions and nuclear fragmentation. The cytoplasmic component of some cells had formed apoptotic bodies, along with a folded nuclear membrane, a tight cytoplasm, and concentrated organelles. The cytoplasmic membranes of the mitochondria were damaged, the crest was fractured, and its density had been reduced and was gathered together by some of the nuclear chromatin, which showed a ring or half shape (Figure 3b) [29]. In the YG group, organelle membrane and endoplasmic reticulum were still intact; the mitochondria were normal and showed a ridge (Figure 3c). The SYG group (Figure 3d) showed a complete cell membrane, clear nucleus, and rough endoplasmic reticulum, plus the mitochondria with the ribosomes next to the nucleus. These results indicated that the YG and the SYG were effective in preventing radiation damage to spleen cells with oxidative stress. Furthermore, the SYG showed a dose-response relationship.

### 3.8. Effects on Proliferation of ConA

Lymphocyte immune response is an important function of cellular components, which can be a direct reflection of the conversion capacity of immune cells [30]. The effect of the YG and the SYG on the proliferation of ConA with irradiation is shown in Table 4. An apparent marked decrease of spleen cell proliferation occurred in the irradiated mice (*p* < 0.05) compared to the untreated mice. This decrease showed that the immune system activity had been injured with irradiation and the ConA induced lymphocyte transformation was inhibited. Although the spleen cell proliferation ability treatment with YG or SYG did not reach that of the untreated. With the administration of YG or SYG, the spleen cell proliferation ability improved compared with the irradiated mice. Moreover, the irradiated mice treated with SYG had the same effect on the spleen cell proliferation ability compared with the YG treatment.

### 3.9. Effects on the Activities of SOD, CAT and Levels of MDA in the Spleen

Duan and Li previously measured lipid oxidative damage as shown by serum lipoperoxide (LPO) levels and superoxide dismutase (SOD) activity [31]. Malondialdehyde (MDA), catalase (CAT), together with SOD are the body's main antioxidant enzyme systems. Not only do they prevent damage caused by the synergistic effect of reactive oxygen species, but they also have a protective role. MDA is a secondary product of lipid peroxidation, and a marker of tissue damage [32]. Lee et al. showed that the increase in CAT activity can eliminate damage caused by hydrogen peroxide’s effect on the body's balance of the redox system [33]. Wu et al. has shown that the serum and the heart from polysaccharide-treatments of aged mice showed lower levels of MDA and higher activities of SOD than those found in untreated aged mice [34].

The effect of the YG and the SYG on the SOD, CAT, and levels of MDA activities in the mice spleen is shown in Table 5. A noticeable marked increase in MDA activity and a significant decrease (*p* < 0.05) of antioxidant enzyme activity (CAT) was observed in the spleen with the irradiated mice compared to the untreated mice. Compared with the irradiated mice, the mice treatment with YG and SYG did not significantly reduce the level of MDA, particularly in the YG group. But the administration of SYG increased the activity of both the CAT and the SOD enzymatic antioxidants in the spleen compared with those from the irradiated mice. Moreover, the mice treated with SYG increased the activity of the enzymes CAT and the SOD in the spleen better those mice treatment with YG.

## 4. Conclusions

The purpose of this study was to study the possibility of obtaining new or robust pharmacological agents with possible therapeutic use using chemical modifications. The research supports the hypothesis that sulfated yeast glucans have significant therapeutic potential. Using RSM simplified the optimization of the temperature, time, and the solvent-solid ratio. Sulfating can improve the water solubility of previously relatively insoluble polysaccharide fractions. The SYG was shown to be better at preventing radiation-induced oxidative damage compared to the untreated YG. Previous research from this laboratory also showed that the immune-enhancing efficacy of sulfated polysaccharides were much better than those of non-modified polysaccharides [35]. Other bioactivities of SYG should also be studied.

## Figures and Tables

**Table 1:** Three factor central composite design matrix and the response values for DS of SYG.

Run	X_1_ Ratio of DMF and HClSO_3_	X_2_ Temperature (°C)	X_3_ Time (h)	DS
1	8	60	2.5	0.454
2	6	50	2.5	0.746
3	8	50	3	0.487
4	8	40	2.5	0.426
5	6	60	2	0.514
6	6	50	2.5	0.717
7	6	50	2.5	0.727
8	6	40	2	0.648
9	6	50	2.5	0.727
10	6	50	2.5	0.732
11	4	50	2	0.446
12	8	50	2	0.493
13	4	60	2.5	0.593
14	4	40	2.5	0.621
15	4	50	3	0.106
16	6	40	3	0.0461
17	6	60	3	0.424

**Table 2:** Analysis of variance (ANOVA) of the regression parameters.

Constant	Coefficient	Standard Deviation	*F*-Value	*p*-Value
Model	0.73	0.05	4.75	0.0260
X_1_	0.012	0.04	0.080	0.7849
X_2_	0.031	0.04	0.55	0.4828
X_3_	−0.13	0.04	9.87	0.0164 *
X_1_X_2_	0.014	0.06	0.056	0.0817
X_1_ X_3_	0.084	0.06	2.06	0.1545
X_2_ X_3_	0.13	0.06	4.81	0.0048 *
X_1_^2^	−0.12	0.06	4.13	0.8190
X_2_^2^	−0.091	0.06	2.55	0.1945
X_3_^2^	−0.23	0.06	16.51	0.0643

* Significant at *p* < 0.05.

**Table 3:** Effects on body weight (BW) and the immune organ indexes of mice radiated using ^60^Co (*n =* 6).

Group	Dose (mg/kg/d)	Initial BW (g)	Final BW (g)	Spleen Index (%)	Thymus Index (%)
Control	0	23.7 ± 1.0	30.5 ± 1.5	0.43 ± 0.04	0.18 ± 0.05
Irradiated mice	6 Gy	24.5 ± 0.3 ^a^	28.2 ± 3.9 ^a^	0.16 ± 0.01 ^a^	0.08 ± 0.01 ^a^
YG	50 + 6 Gy	25.0 ± 2.3 ^a^	30.0 ± 1.6 ^a^	0.18 ± 0.03 ^a^	0.11 ± 0.03 ^a^
SYG	50 + 6 Gy	24.8 ± 0.0 ^a^	30.1 ± 3.3 ^a^	0.21 ± 0.02 ^a,^^c^	0.14 ± 0.03 ^a,^^c^

Results are means ± SD of six measurements. ^a^ *p* < 0.05, compared with the control; ^c^ *p* < 0.05, compared with the Irradiated mice.

**Table 4:** Effects of SYG on the splenocyte proliferation ability of mice radiated with ^60^Co (*n =* 6).

Group	Dose (mg/kg/d)	ConA(%)
Control	0	12.0 ± 0.3
Irradiated mice	6 Gy	9.7 ± 0.1 ^a^
YG	50 + 6 Gy	10.3 ± 0.2 ^a,^^c^
SYG	50 + 6 Gy	10.4 ± 0.1 ^a,^^c^

Results are means ± SD of six measurements. ^a^ *p* < 0.05, compared with the control; ^c^ *p* < 0.05, compared with the Irradiated mice.

**Table 5:** Effects on activities of SOD, CAT and MDA in spleen of mice radiated with ^60^Co (*n =* 6).

Group	Dose (mg/kg/d)	MDA (nmol/mL)	CAT (U/mL)	SOD (U/mL)
Control	0	1.4 ± 0.4	30 ± 2	123 ± 5
Irradiated mice	6 Gy	1.9 ± 0.3 ^a^	17 ± 3 ^b^	84 ± 4 ^b,c^
YG	50 + 6 Gy	2.1 ± 0.3 ^a^	20 ± 2 ^b^	115 ± 3 ^a^
SYG	50 + 6 Gy	1.9 ± 0.5 ^a^	26 ± 1 ^a,c^	117 ± 6 ^a^

Results are means ± SD of six measurements. ^a^ *p* < 0.05, ^b^ *p* < 0.01; compared with the control; ^c^ *p* < 0.05, compared with the irradiated mice.
